# Organ motion in linac-based SBRT for glottic cancer

**DOI:** 10.1186/s13014-021-01833-2

**Published:** 2021-06-12

**Authors:** Annarita Perillo, Valeria Landoni, Alessia Farneti, Giuseppe Sanguineti

**Affiliations:** 1 Department of Radiation Oncology, IRCSS Regina Elena National Cancer Institute, Rome, Italy; 2 Department of Medical Physics, IRCSS Regina Elena National Cancer Institute, Via Elio Chianesi 53, 00144 Rome, Italy

**Keywords:** Organ motion, Daily CBCT imaging, SBRT, Glottic cancer

## Abstract

**Purpose:**

The purpose of this study is to evaluate inter- and intra-fraction organ motion as well as to quantify clinical target volume (CTV) to planning target volume (PTV) margins to be adopted in the stereotactic treatment of early stage glottic cancer.

**Methods and materials:**

Stereotactic body radiotherapy (SBRT) to 36 Gy in 3 fractions was administered to 23 patients with early glottic cancer T1N0M0. Patients were irradiated with a volumetric intensity modulated arc technique delivered with 6 MV FFF energy. Each patient underwent a pre-treatment cone beam computed tomography (CBCT) to correct the setup based on the thyroid cartilage position. Imaging was repeated if displacement exceeded 2 mm in any direction. CBCT imaging was also performed after each treatment arc as well as at the end of the delivery. Swallowing was allowed only during the beam-off time between arcs. CBCT images were reviewed to evaluate inter- and intra-fraction organ motion. The relationships between selected treatment characteristics, both beam-on and delivery times as well as organ motion were investigated.

**Results:**

For the population systematic (Ʃ) and random (σ) inter-fraction errors were 0.9, 1.3 and 0.6 mm and 1.1, 1.3 and 0.7 mm in the left-right (X), cranio-caudal (Y) and antero-posterior (Z) directions, respectively. From the analysis of CBCT images acquired after treatment, systematic (Ʃ) and random (σ) intra-fraction errors resulted 0.7, 1.6 and 0.7 mm and 1.0, 1.5 and 0.6 mm in the X, Y and Z directions, respectively. Margins calculated from the intra-fraction errors were 2.4, 5.1 and 2.2 mm in the X, Y and Z directions respectively. A statistically significant difference was found for the displacement in the Z direction between patients irradiated with > 2 arcs versus ≤ 2 arcs, (MW test, *p* = 0.038). When analyzing mean data from CBCT images for the whole treatment, a significant correlation was found between the time of delivery and the three dimensional displacement vector (r = 0.489, *p* = 0.055), the displacement in the Y direction (r = 0.553, *p* = 0.026) and the subsequent margins to be adopted (r = 0.626, *p* = 0.009). Finally, displacements and the subsequent margins to be adopted in Y direction were significantly greater for treatments with more than 2 arcs (MW test *p* = 0.037 and *p* = 0.019, respectively).

**Conclusions:**

In the setting of controlled swallowing during treatment delivery, intra-fraction motion still needs to be taken into account when planning with estimated CTV to PTV margins of 3, 5 and 3 mm in the X, Y and Z directions, respectively. Selected treatments may require additional margins.

## Introduction

Laryngeal cancer is the most frequent cancer of the upper respiratory tract (28 %) affecting over 13,500 patients a year in the United States and causing about 3700 deaths [1]. Approximately 2/3 of larynx tumors occur in the glottic region (60–65%) and are diagnosed at an early stage, T1-2 N0 M0.

The standard treatment for early glottic cancer is surgery (open or endoscopic) or radiotherapy (RT); both approaches provide excellent oncological outcomes with overlapping local control rates at ≈ 90% for T1^2–4^ and ≈ 80 % for T2 lesions [2–5]. RT may allow a slightly better quality of voice than surgery [6] and the recommended doses for T1 lesions of the true vocal cords (TVC) are 63-66 Gy in 28–33 fractions to the whole larynx.

In parallel to technological improvements which have allowed to irradiate the tumor with increasing accuracy and thus to administer higher doses per fraction, hypofractionated schedules have been explored to shorten the overall duration of treatment and reduce treated volumes in order to minimize the risk of morbidity. Al-Mamgani et al. [7] investigated the feasibility of single cord irradiation with intensity-modulated radiation therapy (IMRT) under image guidance (IGRT) to the total dose of 58.08 Gy in 16 fractions (3.63 Gy per fraction). At a median follow-up of 30 months, the local control rate was 100% in 30 patients with cT1a lesions, without significant acute or long-term toxicity. The voice handicap index (VHI) improved significantly over time, from 33 (baseline) to 9.5 after 6 weeks and to 10 at 18 months.

In this setting, the expansion from the Clinical Target volume (CTV) to the Planning Target Volume (PTV) is a delicate trade-off between the needs of having the target volume covered during each treatment session and of sparing the surrounding normal structures such as the contralateral vocal cord and the arytenoids. Swallowing is associated with laryngeal elevation of approximately 2 cm [8]. However, due to breathing, tumor motion may occur even when the patient is not swallowing [9, 10] and this may become an issue when dose coverage has to be achieved in the presence of high dose gradients. In the present study, inter- and intra-fraction motion have been investigated and the appropriateness of adopted margins have been estimated.

## Methods and materials

### SBRT planning and delivery

From January 2017 to August 2020, 23 patients (18 men and 5 women) were enrolled in a prospective, IRB approved (Registry number 897/16), phase II study on SBRT for glottic cancer in stage I at the IRCCS Regina Elena National Cancer Institute in Rome. Most patients were smokers or former smokers and 70% of tumors involved the anterior 2/3 of the true vocal cords; 10 tumors were bilateral.

Patients were prescribed 36 Gy to the portion of the true vocal cord affected by the tumor and 30 Gy to the immediate surrounding TVC volumes, i.e. the volume at risk of microscopic disease, in three fractions every other day as detailed elsewhere [11]. PTVs were obtained by expanding anisotropically the CTVs by 3 mm in the left-right (LR) and anterior-posterior (AP) directions and by 5 mm in the cranio-caudal (CC) direction. Treatment plans were optimized with Eclipse v.5.5 aiming at PTV coverage of V95 > 95 % with a maximum dose < 107%. A volumetric intensity modulated arc with 6MV FFF technique was used at 1400 MU/min maximum dose rate and treatments were delivered with a Varian True Beam linac equipped with a 120 leaf Millenium multileaf collimator. Nineteen patients were irradiated with 2 arcs while 4 patients with 3 or 4 arcs.

**Fig. 1:**
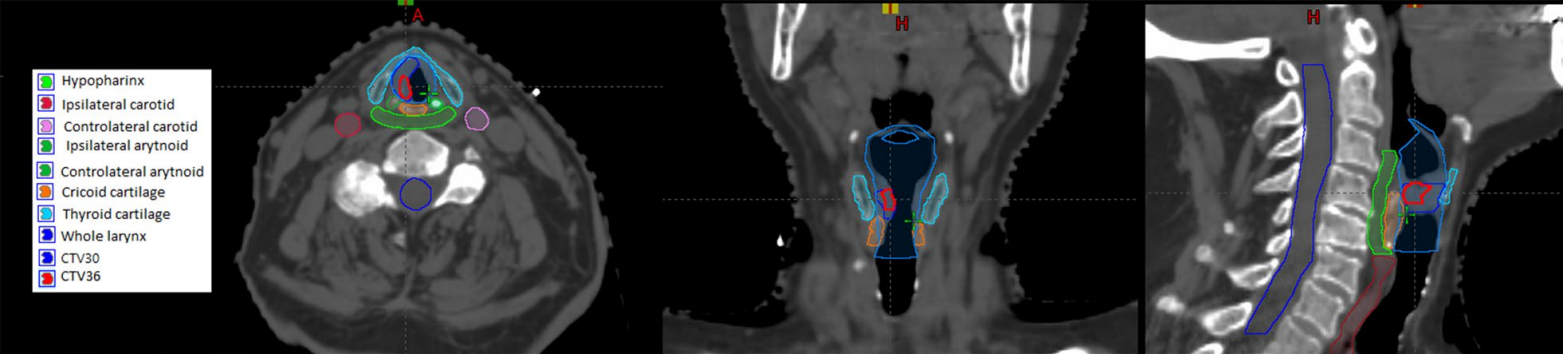
Target and organs at risk as delineated on CT images

All patients underwent both a diagnostic microlaryngoscopy (MLSD) and a larynx MRI to identify the site of the disease for contouring. Afterwards, they underwent planning CT (plCT) in the treatment position with a thermoplastic mask with bite block acquired with 1.25 mm spacing. Figure 1 shows the volumes of interest, the target and selected organs at risks as delineated on plCT images.

Patients were verbally instructed not to swallow during CT and CBCT acquisitions as well as treatment delivery, swallowing was only allowed in the beam off time between treatment arcs. A copy of the protocol is available upon request to the corresponding author.

### CBCT image acquisition and data analysis

**Fig. 2:**
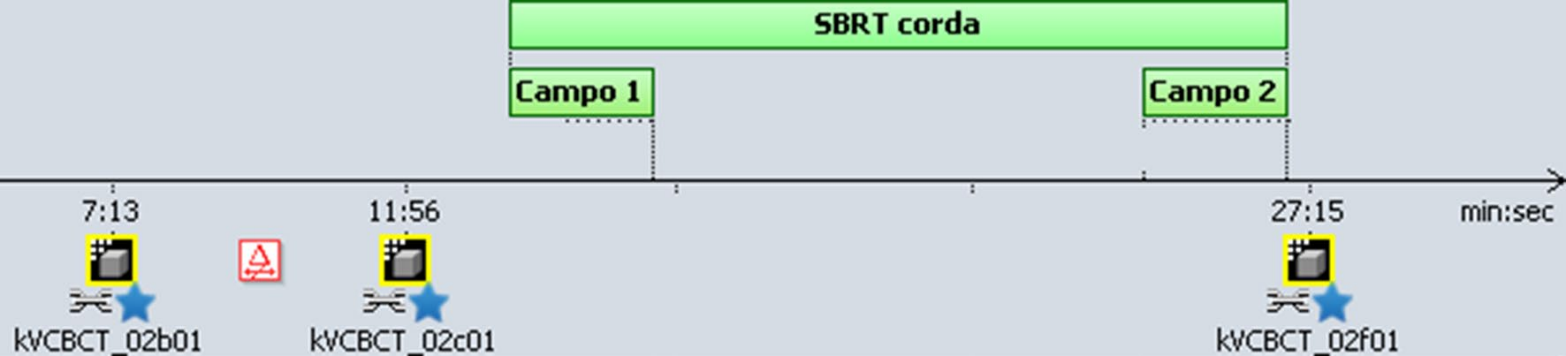
The sequence of image acquisition: a CBCTsetup is acquired, eventually setup corrections are applied, CBCTpre is acquired, at the completion of the irradiation CBCTpost is acquired

A first CBCT image (CBCTsetup) with swallowing control was acquired for each patient and for each treatment session and registered on the planning CT, using the thyroid cartilage for image matching. In case of a setup error > 1 mm and up to 2 mm in any direction, the required couch correction was applied automatically and the patient treated; in case of errors > 2 mm, a new CBCT image was acquired after couch correction. Therefore, the CBCT before treatment delivery (CBCTpre) was considered to be the CBCTsetup when the setup error was within 2 mm; otherwise the repeated CBCT was used as CBCTpre. At the completion of treatment, a final CBCT image was performed (CBCTpost), always maintaining control of swallowing. All procedures were performed as part of routine care by therapists under the supervision of the treating radiation oncologist. The sequence of image acquisition is shown in Fig. 2.

The displacement values indicated by the CBCT imaging immediately before treatment (CBCT setup or CBCTpre) and immediately after delivery (CBCTpost) were recorded for each patient by matching the position of the thyroid cartilage; data of the displacement in the three directions (left-right, cranio-caudal and antero-posterior, respectively X, Y and Z) were then extracted.

The systematic error S_p,i_ (average deviation in each direction of the three treatment sessions) and random error σ_p,i_ (standard deviation of displacements in each direction of the three treatment sessions) were calculated, where p refers to the patient and i to the session.

For the whole population the set-up variations were summarized by µ_i_ (the average of all systematic errors, S_p,i_), Ʃ_i_ (the standard deviation of all systematic errors S_p,i_) and σ_i_ (the root mean square of all random errors σ_i_). By applying the Van Herk formula [12, 13], CTV to PTV margins were calculated in each direction i : M_i_ = 2.5 · Ʃ_i_ + 0.7 · σ_i_.

For each patient and each session, the total vector in space for the displacement, 3D vector, was calculated as the square root of the quadratic sum of the displacements in the three directions X, Y and Z.

Beam on time of each arc and beam delivery time of each session were recorded for each patient.

The non-parametric Mann-Whitney test was used to assess significant differences between variables. All statistical analyses were performed with MedCalc v8.1.0.0 software.

## Results

69 CBCTsetup were performed, but a repeated CBCT was necessary after correction in 20 patients. Therefore, CBCTpre include 49 CBCTsetup and 20 repeated CBCT (rCBCTsetup).

Unfortunately, due to the lack of co-registration of CBCT acquired at the end of the treatment session, treatment iosocenter position was not avalilable for the offline review, thus only 43 CBCTpost for 16 patients are available for analysis. All CBCT images were acquired and recorded by Varian TrueBeam system.

Inter-fraction motion was determined by matching the CBCTpre (either CBCTsetup or rCBCTsetup) to the original planning CT.

Systematic (Ʃ) and random (σ) inter-fraction errors were 0.9, 1.3 and 0.6 mm and 1.1, 1.3 and 0.7 mm in left-right (X), cranio-caudal (Y) and antero-posterior (Z), respectively. Inter-fraction displacements are reported in Table 1.

**Table 1:** Interfraction errors measured by matching CBCT setup/pre with CT planning

Interfraction thyroid cartilage motion (mm)			
*Error type*	X	Y	Z
µ	− 0.1	− 0.3	-0.6
∑	0.9	1.3	0.6
σ	1.1	1.3	0.7

∑, systematic error; σ, random error; µ, average of errors; X, left-right; Y, cranio-caudal; Z, antero-posterior; CBCTsetup, first scan made after setting up the patient on the treatment couch with the room lasers; CBCTpre, second scan possibly performed immediately before treatment

Likewise, intra-fraction motion was determined by matching 43 CBCTpost of 16 patients to the CBCTpre. From the analysis of CBCT images acquired after treatment systematic (Ʃ) and random (σ) intra-fraction errors resulted 0.7, 1.6 and 0.7 mm and 1.0, 1.5 and 0.6 mm in X, Y and Z directions, respectively. Therefore, margins were estimated to be 2.4, 5.1 and 2.2 mm in the X, Y and Z directions, respectively. Intra-fraction errors and margins are reported in Table 2.

**Table 2:** Intrafraction errors measured by matching CBCTpost with CBCT setup/pre and resulting margins

Intrafraction thyroid cartilage motion (mm)			
*Error type*	X	Y	Z
µ	0.1	0.6	0.6
∑	0.7	1.6	0.7
σ	1.0	1.5	0.6
M	2.4	5.1	2.2

All other abbreviations are as in Table 1

CBCTpost, scan made at the end of treatment; M, CTV-PTV margins

Out of 16 patients with CBCTpost, 8 (50 %) had intra-fraction displacements always within the estimated CTV-PTV margins. Of the remaining 8 patients with at least one sessions exceeding CTV-PTV margins, displacement was observed 4, 5 and 6 times in the X, Y and Z directions, respectively. The largest deviations in the X and Z directions were 7.2 and 4.9 mm, respectively, while the largest displacement (9.2mm) was observed in the CC direction, with a systematic error of 4.5 mm and a random error of 4.1 mm. Of note, the higher values were observed in the Y direction resulting in margins up to 5.2 mm.

**Fig. 3:**
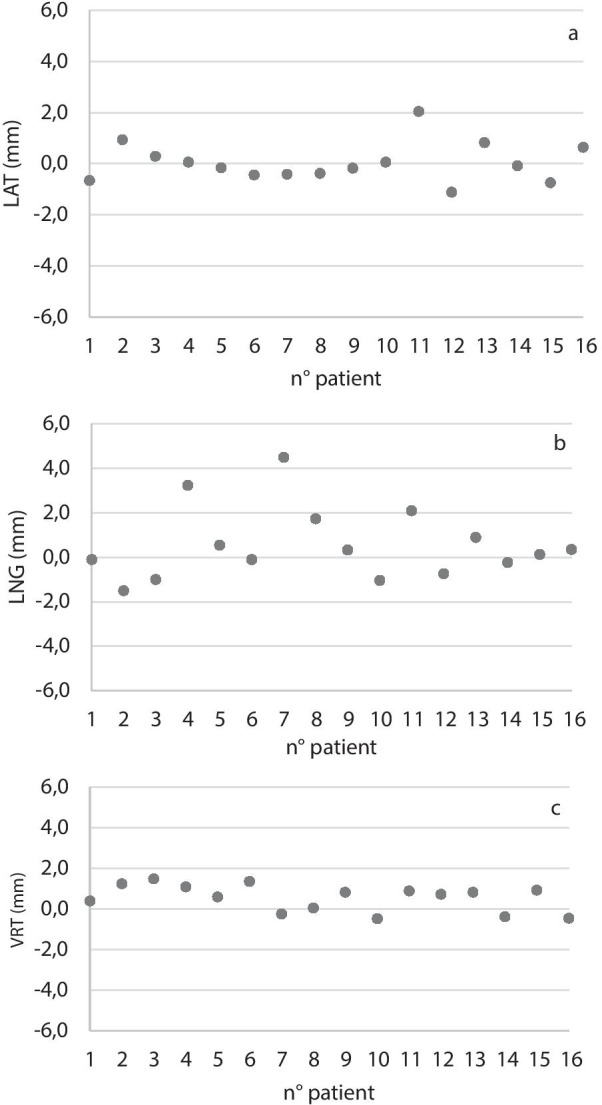
Systematic intra-fraction displacement of the thyroid cartilage in the three directions evaluated for each patient at the end of treatment; **a** lateral direction, **b** longitudinal direction, **c** vertical direction

In Fig. 3, the intra-fraction systematic error for each patient is shown. The deviations in the lateral and vertical directions were very small (ranging from − 1.1 to 2.1 mm and from − 0.5 to 1.5 mm, respectively) while larger values have been recorded in the longitudinal direction (from − 1.5 to 4.5 mm).

**Fig. 4:**
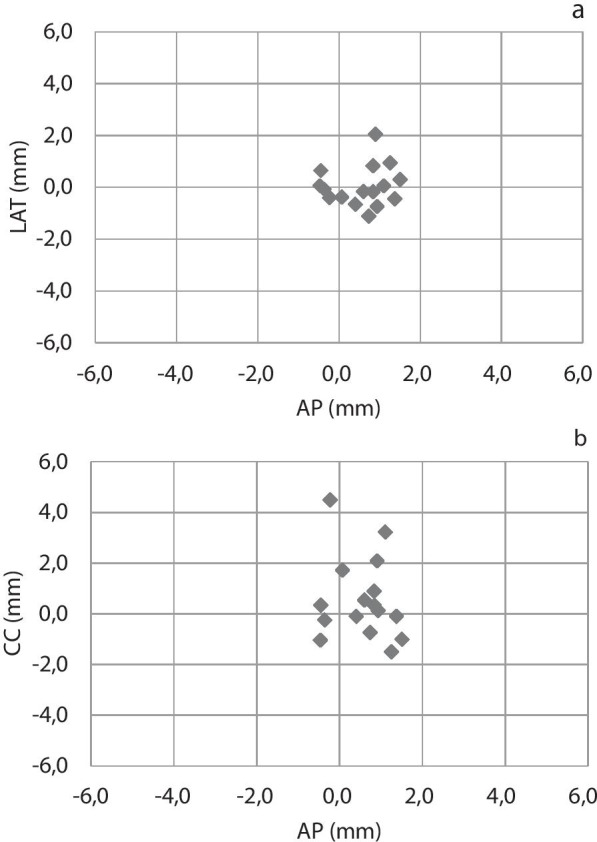
Systematic intra-fraction error (displacements in each direction averaged in the three treatment patient sessions); **a** lateral versus antero-posterior, **b** cranio-caudal versus antero-posterior

A similar behavior was observed within the entire population as shown in Fig. 4: smaller displacements in the latero-lateral and anteroposterior directions and larger deviations in the cranio-caudal direction.

The median 3D vector was 2.7 mm (range 0.0–9.6). The median treatment delivery time was 13.3 min (range 8.8–87.0) while the median beam on time was 2.1 min (range 0.9–4.5).

When analyzing mean data for the whole treatment, a significant correlation was found between the delivery time and the three dimensional displacement vector (r = 0.489, *p* = 0.055), the displacement in the Y direction (r = 0.553, *p* = 0.026) and the subsequent margins to be adopted (r = 0.626, *p* = 0.009) with patients undergoing shorter treatments showing greater compliance. No correlation was found between the mean 3D vector and the beam-on time which is very similar among patients.

A statistically significant difference was found for the displacement in the Z direction between patients irradiated with > 2 arcs or ≤ 2 arcs *p* = 0.038).

Moreover, both the displacement and the subsequent margins to be adopted in Y direction are significantly greater in treatments with more than 2 arcs (MW test *p* = 0.037 and *p* = 0.019, respectively).

No correlation was found between the volumes of CTV 30 and CTV 36 and other variables that are very similar among the population: median 1.2 cc (range 0.3–5.2 cc) and 0.4 cc (range 0.1–2.1 cc), respectively.

## Discussion

The larynx is a rather mobile organ due to swallowing, breathing, and phonation. Glottic intra-fraction motion in the cranio-caudal direction is linked to the intrinsic mobility of the larynx that cannot be prevented by the thermoplastic mask [8]. The effect of swallowing on laryngeal position is well known from video-fluoroscopy studies that have shown maximum displacements in the longitudinal direction between 20 and 25 mm [14–16].

In a recent phase II study, Bo Zhao et al. [17] treated 10 patients with early stage glottic cancer with SBRT (42.5 Gy in 5 fractions) and used a surface tracking tool to monitor organ motion. A small region of the immobilization mask was manually opened to allow surface tracking to monitor the unexpected swallowing events and related glottic displacements. Pre-treatment and intra-fraction CBCTs were acquired to verify internal anatomy. Patients were verbally instructed not to swallow during treatment and a Motion Management Interface (MMI) system recorded changes in the surface anatomy of the anterior neck region. When the detected movement was within the threshold value of 3 mm, the beam was delivered instead, if displacement exceeded the stated limit, delivery was automatically stopped. Considering the entire duration of treatment (i.e. 5 fractions), including the time in which patients were not instructed to hold their swallowing, the frequency and the duration of swallowing varied between patients and also between fractions (6.6 ± 5.2 times for fraction and 3.9 ± 2.5 s for swallowing respectively) with a mean peak amplitude of each swallow of 5.8 ± 3.8 mm above baseline, mainly in the longitudinal direction. However, swallowing events were relevant only during the delivery time during which they occurred less frequently due to verbal instructions that reduced them to 0.8 ± 1.4 times per fraction. Verbal instruction reduced also the magnitude of motion from 3.4 to 2.7 mm (95th percentile). Moreover, associating tracking resulted in a further reduction down to 2.3 mm; displacement was reduced from 1.35 ± 1.53 to 1.07 ± 0.73 mm by both methods. The authors concluded that the two methods have led to very similar results for most patients so verbal instruction alone may be sufficient for appropriate control of organ motion for probably most patients.

Paulson et al. [18] used cine MRI to assess the internal margin size based on the actual deglutition-induced tumor motion in head and neck patients. Compared to a video-fluoroscopy and endoscopy, the gold standard for assessing aero-digestive tract motion and function, cine MRI is non ionizing and completely non-invasive method assessing swallowing events by dynamic changes in MR signal intensities caused by anatomical structures. They observed a deglutition induced displacement and a resting displacement. The first is always greater than the second and in both cases the largest displacement was observed in the Y direction. The resting displacement indicates that a motion occurs even when the patient is not swallowing. Also Bradley et al. [19] quantified frequency of swallowing and tumor and normal structure displacements during deglutition using dynamic MRI and determined PTV margins to account for resting and deglutition-induced displacements in head and neck patients. They found a measurable mean maximum resting displacement for GTV indicating that movements occur even in the absence of swallowing. Intra-fraction motion occurs because of respiration and tongue movements. The data of this study suggest that a PTV margin is required to account for tumor motion also during a non-swallowing state and it must be larger than the setup error because of the component of resting tumor motion.

Therefore, we elected to verbally instruct patients not to swallow during both imaging and dose delivery. Interestingly, our margins (2.4, 5.1 and 2.2 mm in the X, Y and Z directions) are very similar to the ones estimated by Kwa et al. [20] (1.6, 4.3 and 2.2 in the X, Y and Z directions, respectively) who adopted the same strategy of withholding swallowing.

As a matter of fact, in our study we assumed that swallowing did not occur during dose delivery. Moreover, we choose the thyroid cartilage as a surrogate for vocal cord position, because it adheres directly to the involved vocal cord [7], even if this does not address the issue of residual motion due to breathing. To fully take into account the latter, Sher et al. [21] in a phase I Fractional Dose-Escalation Study for Early-Stage Glottic Larynx Cancer simulated patients by 4DCT imaging and applied margin sizes of 2 mm and 3 mm to the ITV and CTV to obtain CTV and PTV, respectively. However, Al-Mamgani et al. [7], report that, in their experience, the maximum intensity projection of the vocal cord was not fully included in the initial CTV contours only in a limited number of patients, and deviations were usually below 1 mm. Therefore, the lack of correction for this residual motion would have had a minimal impact on dose coverage. Also Osman et al. conducted a study to quantify intrafraction motion of the vocal cords due to respiration using 4D-CT; they found that respiratory motion compensation techniques, such as active breathing control, gating, or tumor tracking, do not seem to enhanced significantly the feasibility of single vocal cords irradiation [22].

In our study, each treatment session was relatively short (13.3 min, range 8.8–87.0), even though we found a significant correlation between the delivery time and the displacement size. In fact, when patients were treated with more than 2 arcs, the displacement and therefore the margins in Y direction were significantly larger than in patients treated with 2 arcs probably due to a relaxation of the larynx with passing time. This would support planning towards quicker and fewer arc treatments.

One limitation of this study is the fact that rotational set-up errors were not corrected. Moreover, deformation was not considered when calculating margins though the geometric accuracy of deformable image registration in this setting should be discussed [23]. Hence, an accurate evaluation of margins to be adopted seems to be the most reliable strategy to assure target coverage and a diminishing of margins should be eventually adopted with great caution.

## Conclusions

SBRT of early stage glottic cancer seems to be a viable therapeutic option in the future despite being dosimetrically challenging. Intra-fraction motion can be substantial; therefore, it must be considered when evaluating the CTV-PTV margins for planning. In our study, the estimated a posteriori CTV-PTV margins of 2.7, 5.2 and 2.1 mm in LR, CC and AP directions, respectively are similar to those indicated by the treatment protocol and appear sufficient and adequate for a good target coverage. New techniques such as swallowing and gating could further reduce the intra-fraction motion but still no evidence leads to smaller margins. Further studies may help instead, to validate techniques of gated delivery that could allow to safely treat patients in highly hypo-fractionated regimens.

## Data Availability

Data is available at IRCCS Regina Elena National Cancer Institute.

## Abbreviations

CBCT: Cone beam computed tomography
RT: Radiotherapy
TVC: True vocal cord
IMRT: Intensity modulated radiation therapy
SBRT: Stereo body radiation therapy
IGRT: Image guided radiation therapy
VHI: Voice handicap index
CTV: Clinical target volume
PTV: Planning target volume
LR: Lateral
AP: Antero-posterior
CC: Cranio-caudal
MLSD: Diagnostic microlaryngoscopy
plCT: Planning computed tomography
MMI: Motion management interface

## References

[1] Siegel RL, Miller KD, Fedewa SA, Ahnen DJ, Meester RGS, Barzi A (2017). Colorectal cancer statistics, 2017. CA Cancer J Clin.

[2] Hartl DM, Ferlito A, Brasnu DF, Langendijk JA, Rinaldo A, Silver CE (2011). Evidence-based review of treatment options for patients with glottis cancer. Head Neck.

[3] Mendenhall WM, Werning JW, Hinerman RW, Amdur RJ, Villaret DB (2004). Management of T1-T2 glottic carcinomas. Cancer.

[4] Y VL, Ev S, Tp L, Jong RJB, Jw S, Rossum MA (2012). Functional out comes after radiotherapy or laser surgery in early glottic carcinoma: a systematic review. Head Neck.

[5] Sjögren EV, Langeveld TPM, Baatenburg de Jong RJ (2008). Clinical outcome of T1 glottic carcinoma since the introduction of endoscopic CO2 laser surgery as treatment option. Head Neck.

[6] Aaltonen LM, Rautiainen N, Sellman J, Saarilahti K, Makitie A, Rihkanen H (2014). Voice quality 0after treatment of early vocal cord cancer: a randomized trial comparing laser surgery with radiation therapy. Int J Radiationoncol Biol Phys.

[7] Al-Mamgani A, Kwa SLS, Tans L, Moring M, Fransen D, Mehilal R (2015). Single vocal cord irradiation: Image guided intensity modulated hypofractionated radiation therapy for T1a glott ic cancer: early clinical results. Int J Radiat Oncol Biol Phys.

[8] Hamlet S, Ezzell G, Aref A (1994). Larynx motion associated with swallowing during radiation therapy. Int J Radiat Oncol Biol Phys.

[9] Bradley JA, Paulson ES, Ahunbay E, Schultz C, Li A, Wang D (2011). Dynamic MRI analysis of tumor and organ motion during rest and deglutition and margin assessment for radiotherapy of head and neck cancer. Int J Radiat Oncol Biol Phys.

[10] Bruijnen T, Stemkens B, Terhaard CHJ, Lagendijk JJW, Raaijmakers CPJ, Tijssen RHN (2019). Intrafraction motion quantification and planning target volume margin determination of head-and-neck tumors using cine magnetic resonance imaging. Radiother Oncol.

[11] Sanguineti G, Pellini R, Vidiri A, Marzi S, D’Urso P, Terrenato I, et al. Stereotactic body radiotherapy for T1 glottic cancer: dosimetric data in 27 consecutive patients. Tumori 2021:3008916211000440. 10.1177/03008916211000440 33821713

[12] van Herk M, Remeijer P, Rasch C, Lebesque JV (2000). The probability of correct target dosage: dose-population histograms for deriving treatment margins in radiotherapy. Int J Radiat Oncol Biol Phys.

[13] Stroom JC, de Boer HC, Huizenga H, Visser AG (1999). Inclusion of geometrical uncertainties in radiotherapy treatment planning by means of coverage probability. Int J Radiat Oncol Biol Phys.

[14] Dantas RO, Kern MK, Massey BT, Dodds WJ, Kahrilas PJ, Brasseur JG (1990). Effect of swallowed bolus variables on oral and pharyngeal phases of swallowing. Am J Physiol.

[15] Jacob P, Kahrilas PJ, Logemann JA, Shah V, Ha T (1989). Upper esophageal sphincter opening and modulation during swallowing. Gastroenterology.

[16] Leonard RJ, Kendall KA, McKenzie S, Gonçalves MI, Walker A (2000). Structural displacements in normal swallowing: a videofluoroscopic study. Dysphagia.

[17] Zhao B, Park YK, Gu X, Reynolds R, Timmerman R, Sher DJ (2020). Surface guided motion management in glottic larynx stereotactic body radiation therapy. Radiother Oncol.

[18] Paulson ES, Bradley JA, Wang D, Ahunbay EE, Schultz C, Li XA (2011). Internal margin assessment using cine MRI analysis of deglutition in head and neck cancer radiotherapy: HNC margin assessment using cine MRI. Med Phys.

[19] Bradley JA, Paulson ES, Ahunbay E, Schultz C, Li XA, Wang D (2011). Dynamic MRI analysis of tumor and organ motion during rest and deglutition and margin assessment for radiotherapy of head-and-neck cancer. Int J Radiat Oncol Biol Phys.

[20] Kwa SLS, Al-Mamgani A, Osman SOS, Gangsaas A, Levendag PC, Heijmen BJM (2015). Inter- and intrafraction target motion in highly focused single vocal cord irradiation of T1a larynx cancer patients. Int J Radiat Oncol Biol Phys.

[21] Sher DJ, Timmerman RD, Nedzi L, Ding C, Pham N-L, Zhao B, Sumer BD (2019). Phase 1 fractional dose-escalation study of equipotent stereotactic radiation therapy regimens for early-stage glottic larynx cancer. Int J Radiat Oncol Biol Phys.

[22] Osman SOS, de Boer HCJ, Heijmen BJM, Levendag PC (2008). Four-dimensional CT analysis of vocal cords mobility for highly focused single vocal cord irradiation. Radiother Oncol J Eur Soc Therap Radiol Oncol.

[23] Schultheiss TE, Tome WA, Orton CG (2012). Point/counterpoint: it is not appropriate to “deform” dose along with deformable image registration in adaptive radiotherapy: point/counterpoint. Med Phys.

